# Utility and Comparative Efficacy of Lok Score, Splenic Stiffness, EVendo Score, and Liaoning Score in Predicting Esophageal Varices in the Pakistani Population

**DOI:** 10.7759/cureus.85098

**Published:** 2025-05-30

**Authors:** Salman Ahsam, Muhammad Aslam, Sanaullah Kalwar, Khaild Tareen, Ali Hyder, Ayesha Shakil, Raja Taha Yaseen Khan, Syeda Maryam Mehdi, Nasir Hassan Luck

**Affiliations:** 1 Gastroenterology, Social Security Hospital, Faisalabad, PAK; 2 Gastroenterology, Madinah Teaching Hospital, Faisalabad, PAK; 3 Gastroenterology and Hepatology, Gambat Institute of Medical Sciences, Gambat, PAK; 4 Gastroenterology and Hepatology, Sheikh Khalifa Bin Zayed Al Nahyan Hospital, Quetta, PAK; 5 Gastroenterology, Chandka Medical College, Shaheed Mohtarma Benazir Bhutto Medical University, Larkana, PAK; 6 Medicine, Dow University of Health Sciences, Civil Hospital Karachi, Karachi, PAK; 7 Hepatogastroenterology, Sindh Institute of Urology and Transplantation, Karachi, PAK; 8 Medicine, Ziauddin University, Karachi, PAK; 9 Gastroenterology, Sindh Institute of Urology and Transplantation, Karachi, PAK

**Keywords:** cirrhosis, esophageal varices, evendo score, liaoning score, lok score, non-invasive prediction, pakistan, splenic stiffness

## Abstract

Background

Esophageal varices (EV) are a critical complication of portal hypertension in cirrhotic patients, with variceal bleeding contributing significantly to morbidity and mortality. Endoscopy remains the gold standard for diagnosis, but its invasive nature and limited availability in low-resource settings necessitate the validation of non-invasive predictive tools. This study aims to compare the diagnostic performance of four non-invasive models, Lok score, splenic stiffness (SS), EVendo score, and Liaoning score, for predicting EV in Pakistani patients with chronic liver disease (CLD).

Methods

A cross-sectional observational study was conducted at the Department of Hepatogastroenterology, Sindh Institute of Urology and Transplantation (SIUT), Karachi, from January to December 2024. A total of 272 cirrhotic patients undergoing screening endoscopy were enrolled. Clinical, laboratory, and radiological data were collected to compute the Lok, EVendo, and Liaoning scores. Splenic stiffness was measured using transient elastography. The presence of EV was confirmed via upper gastrointestinal (GI) endoscopy. Receiver operating characteristic (ROC) curve analysis was used to assess diagnostic accuracy (area under the receiver operating characteristic {AUROC}), and multivariate regression identified independent predictors of high-risk varices (HRV).

Results

EV were present in 72.8% of patients, with high-risk varices in 31.3%. All four scores were significantly elevated in patients with EV. Splenic stiffness demonstrated the highest AUROC (0.858), followed by Lok score (0.804), EVendo score (0.76), and Liaoning score (0.755). EVendo score showed the highest diagnostic accuracy (81.56%) and, along with SS, was an independent predictor of HRV.

Conclusion

Splenic stiffness and EVendo score are reliable non-invasive predictors of esophageal varices and high-risk varices in the Pakistani cirrhotic population. However, further multicentered studies are required to validate our results.

## Introduction

Esophageal varices (EV) are dilated submucosal veins primarily located in the distal esophagus, commonly arising as a severe complication of portal hypertension in cirrhotic patients [1]. The development of EV is a major milestone in the natural history of cirrhosis, often signifying the transition from compensated to decompensated liver disease [2]. The most feared consequence of EV is variceal hemorrhage, which is associated with a high risk of morbidity and mortality, with a first bleeding episode carrying a mortality rate of up to 20% despite advances in therapy [3].

Endoscopic surveillance is the gold standard for diagnosing esophageal varices [4]. However, routine endoscopic screening for all patients with cirrhosis is associated with challenges. Endoscopy is invasive, expensive, resource-intensive, and uncomfortable for the patient [5]. In resource-limited settings such as Pakistan, universal screening is logistically impractical. Moreover, a significant proportion of cirrhotic patients subjected to screening endoscopy are found to have no varices or only low-risk varices that do not require intervention, leading to unnecessary procedures and associated healthcare costs [2].

Recently, multiple non-invasive predictive models and assessment tools have been developed for the prediction of EV [6]. The Lok score, derived from simple laboratory parameters including platelet count, international normalized ratio (INR), bilirubin, and aspartate aminotransferase/alanine aminotransferase (AST/ALT) ratio, was initially developed to predict cirrhosis but has since been repurposed to predict portal hypertension and esophageal varices [7]. Splenic stiffness measurement (SSM), obtained through transient elastography, has emerged as another promising non-invasive marker, correlating more strongly with portal hypertension than liver stiffness in several studies [8]. The EVendo score, a recently validated composite model, integrates liver stiffness, platelet count, and spleen diameter, offering a practical and efficient prediction tool [9,10]. Additionally, the Liaoning score, developed in a Chinese cohort, combines clinical and laboratory parameters and has shown promising results for predicting high-risk varices (HRV) [11].

However, the validation of these scores and tools remains variable across populations due to differences in the liver disease etiologies, demographics, and healthcare settings. Most models are derived from Western or East Asian populations, and their applicability to South Asian, particularly Pakistani, populations remains unclear. Given the high burden of hepatitis-related cirrhosis in Pakistan and the limitations of endoscopic resources, validating and comparing these non-invasive tools for EV prediction in our population are of utmost clinical importance as it provide us with a reliable non-invasive predictor that would allow us selective endoscopic screening, prioritize patients at highest risk, and optimize healthcare resource utilization in Pakistan.

Therefore, the objective of this study was to evaluate and compare the diagnostic performance of four non-invasive models, Lok score, splenic stiffness (SS), EVendo score, and Liaoning score, in predicting the presence of esophageal varices.

## Materials and methods

After the approval from the Ethical Review Committee (ERC) of Sindh Institute of Urology and Transplantation (SIUT) (approval number: 811), this cross-sectional observational study was conducted at the Department of Hepatogastroenterology, Sindh Institute of Urology and Transplantation (SIUT), from January to December 2024. Patients were enrolled consecutively from the outpatient clinics and inpatient services based on predefined eligibility criteria. All the patients aged 18 years and above with a confirmed diagnosis of cirrhosis, as per operational definition, undergoing screening endoscopy for esophageal varices were enrolled in the study (Table 1). Patients were excluded if they had a previous history of variceal bleeding or endoscopic band ligation, evidence of hepatocellular carcinoma, portal vein thrombosis, active systemic infection, incomplete clinical or elastography data, or pregnancy.

**Table 1:** Operational definitions of cirrhosis of the liver, Lok score, EVendo score, and Liaoning score INR, international normalized ratio; AST, aspartate aminotransferase; ALT, alanine aminotransferase

Operational definitions
Cirrhosis of the liver [12]: The patient with liver cirrhosis will be identified on abdominal ultrasound findings. The presence of three or more of the following findings will be considered as the presence of liver cirrhosis: (1) altered echo texture of the liver, (2) irregular margins, (3) spleen size of more than 12 cm, (4) portal vein diameter of more than 12 mm, and (5) the presence of free fluid in the abdomen on ultrasound
Lok score [7]: log odds = −5.56-0.0089 × platelet count (103/mm^3^) + 1.26 × (AST/ALT) + 5.27 × INR; Lok = [exp (log odds)] / [1 + exp (log odds)]
EVendo score [9]: A = (8.5 × INR) + (AST, U/L / 35); B = (platelet count (×10³/µL) / 150) + (BUN (mg/dL) / 20) + (hemoglobin (g/dL) / 15); EVendo score = (A / B) + 1 (if ascites present)
Liaoning score [5,13]: 1.205 + 1.557 × ascites (1 = yes, 0 = no) − 0.008 × platelet count

The sample size was calculated considering an anticipated area under the receiver operating characteristic (AUROC) curve of 0.80 for the best-performing predictive model, with a 5% margin of error and 95% confidence interval (CI). Based on these assumptions, the minimum required sample size was estimated at 200 patients. To compensate for possible incomplete data or dropouts, a total of 272 patients were enrolled in the study.

Data collection procedure

The demographic data were collected from each patient, such as age, gender, the etiology of liver disease, and Child-Pugh classification. Laboratory investigations, including platelet count, serum aspartate aminotransferase (AST), alanine aminotransferase (ALT), total bilirubin, and international normalized ratio (INR), were recorded. Radiological assessments involved measuring splenic stiffness using transient elastography (FibroScan®, Echosens, Paris, France) by a single trained operator with more than five years of experience in performing hepatobiliary imaging. Using the collected clinical and laboratory data, non-invasive predictive models, including Lok score, EVendo score, and Liaoning score, were calculated based on their respective established formulas. All patients subsequently underwent upper gastrointestinal (GI) endoscopy (GIF-190, Olympus, Tokyo, Japan) performed by an experienced endoscopist with more than 10 years of experience in performing upper GI endoscopies, and the presence, absence, and grading of esophageal varices were documented according to Baveno VII guidelines [4].

Data analysis procedure

The data were entered and analyzed using the Statistical Package for Social Sciences (SPSS) version 26 (IBM Corp., Armonk, NY). Continuous variables were presented as mean ± standard deviation, while categorical variables were expressed as frequencies and percentages. Continuous variables were compared using Student's t-test, while categorical variables were analyzed using the chi-square test.

The diagnostic performance of each non-invasive tool, Lok score, splenic stiffness, EVendo score, and Liaoning score, was assessed through a receiver operating characteristic (ROC) curve analysis. The area under the ROC (AUROC) curve was calculated for each predictor to evaluate its accuracy. Sensitivity, specificity, positive predictive value (PPV), and negative predictive value (NPV) were determined for optimal cutoff points of these non-invasive indices. Multivariate logistic regression analysis was performed for the utility of non-invasive indices in predicting HRV. A p-value of less than 0.05 was considered statistically significant for all analyses.

## Results

A total of 272 patients with chronic liver disease (CLD) were enrolled in the study. Out of them, 167 (61.4%) were men. The most common cause of CLD was hepatitis C virus (HCV) infection, observed in 131 (48.2%) patients, followed by hepatitis B virus (HBV) in 59 (21.7%) patients and alcohol-related liver disease, autoimmune hepatitis (AIH), and HBV-hepatitis D virus (HDV) coinfection in 32 (11.7%), 26 (9.6%), and 21 (7.7%) patients, respectively. The mean age of the patients was 43.6 ± 12.4 years. Among these, 198 (72.8%) patients had esophageal varices (EV), and 85 (31.3%) had high-risk varices (HRV). The mean hemoglobin level was 10.8 ± 2.1 g/dL, and the mean platelet count was 74.8 ± 40.3 × 10⁹/L (Table 2).

**Table 2:** Baseline characteristics of the studied population (n = 272) HCV, hepatitis C virus; HBV, hepatitis B virus; AIH, autoimmune hepatitis; HDV, hepatitis D virus; CTP, Child-Turcotte-Pugh; HRV, high-risk varices; TLC, total leucocyte count; PLT, platelet; AST, aspartate aminotransferase; ALT, alanine aminotransferase; INR, international normalized ratio

Variable		n (%)
Gender	Male	167 (61.4)
	Female	105 (38.6)
Cause of chronic liver disease	HCV	131 (48.2)
	HBV	59 (21.7)
	AIH	26 (9.6)
	HBV + HDV coinfection	21 (7.7)
	Alcohol	32 (11.7)
	Unknown	3 (1.1)
CTP class	A	180 (66.2)
	B	92 (33.8)
Esophageal varices	Yes	198 (72.8)
	No	72 (27.2)
HRV	Yes	85 (31.3)
	No	187 (68.7)
Age (years)		43.6 ± 12.4
Hemoglobin (g/dL)		10.8 ± 2.1
TLC (×10⁹/L)		4.5 ± 2.4
PLT (×10⁹/L)		74.8 ± 40.3
AST (IU/L)		59 ± 52
ALT (IU/L)		45 ± 33
Serum albumin (g/L)		3.8 ± 0.63
INR		1.2 ± 0.19
Liaoning score		0.78 ± 0.57
EVendo score		18.5 ± 7.3
Lok score		0.54 ± 0.12
Splenic stiffness (kPa)		41.9 ± 25.1

On comparative analysis, several variables showed significant differences. Patients with varices had significantly lower hemoglobin (10.6 ± 2.1 versus 11.5 ± 2.0 g/dL; p = 0.002) and platelet counts (54 ± 20 versus 129 ± 27 × 10⁹/L; p ≤ 0.001), and higher total leukocyte counts (TLC) were noted in those without varices (6.8 ± 2.1 versus 3.7 ± 2.5; p ≤ 0.001). Liver function markers such as AST (64 ± 58 versus 45 ± 22 IU/L; p ≤ 0.001), ALT (47 ± 37 versus 38 ± 20 IU/L; p = 0.04), serum albumin (3.3 ± 0.6 versus 3.5 ± 0.5 g/L; p = 0.013), and INR (1.2 ± 0.23 versus 1.0 ± 0.1; p ≤ 0.001) were significantly deranged in the EV group (Table 3).

**Table 3:** Comparison of baseline characteristics in predicting the presence of esophageal varices (EV) (n = 272) CTP, Child-Turcotte-Pugh; TLC, total leucocyte count; PLT, platelet; AST, aspartate aminotransferase; ALT, alanine aminotransferase; INR, international normalized ratio

Variable		Esophageal varices		P-value
		Present (N = 198)	Absent (N = 72)	
Gender	Male	114 (57.1)	53 (64)	0.034
	Female	84 (42.9)	21 (36)	
CTP class	A	129 (55.9)	51 (71.8)	0.559
	B	69 (44.1)	23 (28.2)	
Age (years)		42.8 ± 11.5	45.8 ± 14.6	0.089
Hemoglobin (g/dL)		10.6 ± 2.1	11.5 ± 2.0	0.002
TLC (×10⁹/L)		3.7 ± 2.5	6.8 ± 2.1	≤0.001
PLT (×10⁹/L)		54 ± 20	129 ± 27	≤0.001
AST (IU/L)		64 ± 58	45 ± 22	≤0.001
ALT (IU/L)		47 ± 37	38 ± 20	0.04
Serum albumin (g/L)		3.3 ± 0.6	3.5 ± 0.5	0.013
INR		1.2 ± 0.23	1.0 ± 0.1	≤0.001
Splenic stiffness (kPa)		48.1 ± 26.1	25.1 ± 10.8	≤0.001
EVendo score		20.1 ± 7.9	14.3 ± 2.2	≤0.001
Liaoning score		0.88 ± 0.49	0.52 ± 0.67	≤0.001
Lok score		0.57 ± 0.1	0.46 ± 0.14	≤0.001

In terms of non-invasive predictive scores, all four models, EVendo score, Lok score, Liaoning score, and splenic stiffness, were significantly higher in patients with esophageal varices. The EVendo score was notably higher in the EV group (20.1 ± 7.9) compared to those without EV (14.3 ± 2.2; p ≤ 0.001). Similarly, the Lok score (0.57 ± 0.1 versus 0.46 ± 0.14; p ≤ 0.001), Liaoning score (0.88 ± 0.49 versus 0.52 ± 0.67; p ≤ 0.001), and splenic stiffness (48.1 ± 26.1 versus 25.1 ± 10.8 kPa; p ≤ 0.001) were significantly elevated in patients with varices (Table 3).

**Figure 1:**
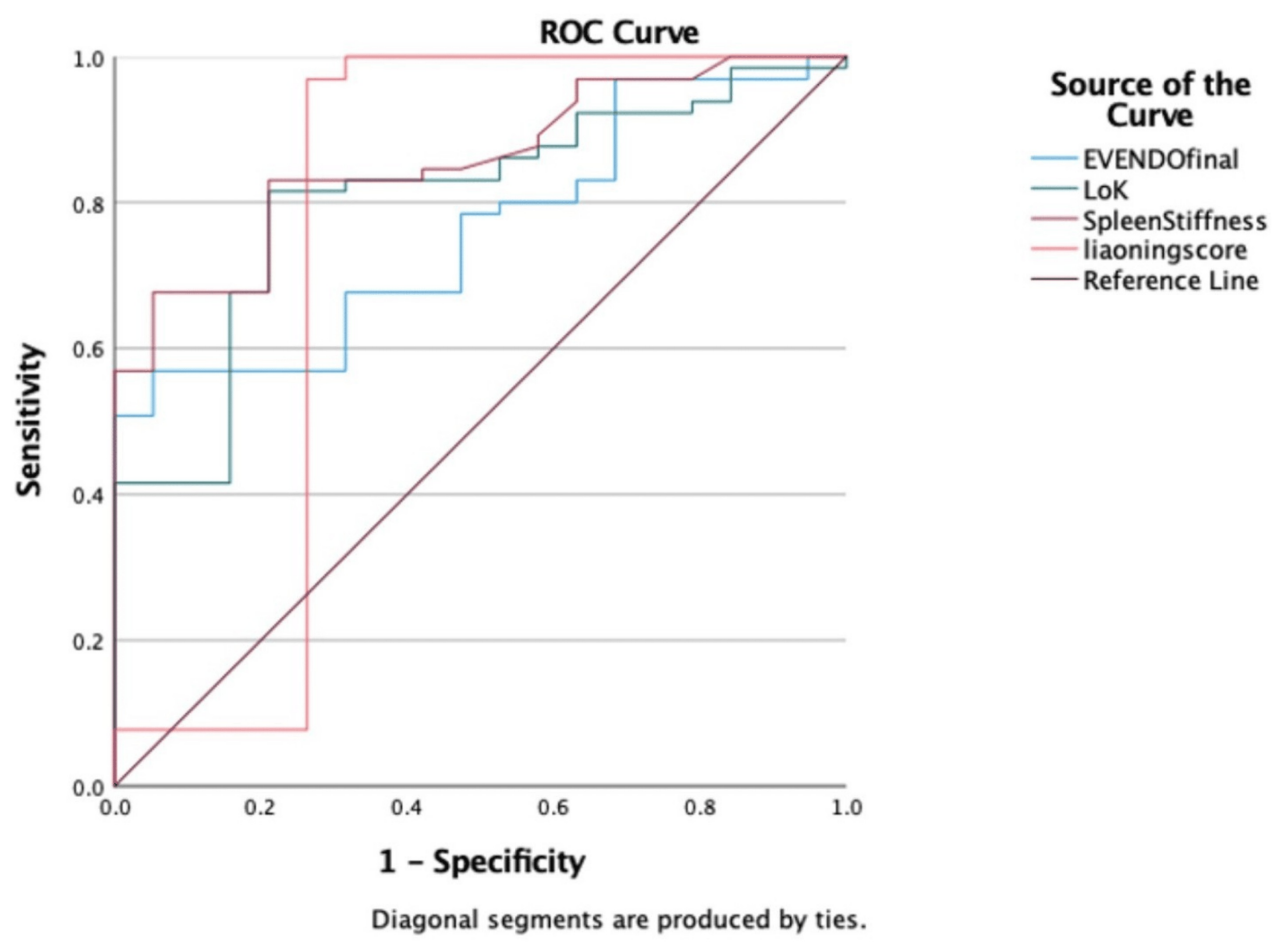
AUROC for Lok score, EVendo score, Liaoning score, and SS in predicting esophageal varices was 0.804 (p ≤ 0.001), 0.76 (p ≤ 0.001), 0.755 (p ≤ 0.001), and 0.858 (p ≤ 0.001), respectively AUROC, area under the receiver operating characteristic; SS, splenic stiffness; ROC, receiver operating characteristic

Area under the receiver operating characteristic (AUROC) curve obtained for all these non-invasive scores revealed that splenic stiffness had the highest AUROC (0.858) for predicting EV, followed by Lok score (0.804), EVendo score (0.76), and Liaoning score (0.755) (Figure 1). The EVendo score at a cutoff of ≥14.5 demonstrated a high sensitivity of 92.94% and specificity of 71.88%, with a diagnostic accuracy of 81.56%. The Lok score, with a threshold of ≥0.55, showed similar sensitivity (92.86%) but lower specificity (58.98%), resulting in a diagnostic accuracy of 58.24%. Liaoning score with a cutoff of <0.55 achieved 77.38% sensitivity and 80.47% specificity, with the highest diagnostic accuracy among all (79.71%). Splenic stiffness at a cutoff of ≥31 kPa yielded 89.29% sensitivity but a lower specificity of 66.88%, with an overall diagnostic accuracy of 76.47% (Table 4).

**Table 4:** Diagnostic accuracy of EVendo score, Lok score, Liaoning score, and splenic stiffness in predicting EV EV, esophageal varices; AUROC, area under the receiver operating characteristic; PPV, positive predictive value; NPV, negative predictive value

Predictive model	AUROC	P-value	Cutoff	Sensitivity	Specificity	PPV	NPV	Diagnostic accuracy
EVendo score	0.76	≤0.01	≥14.5	92.94%	71.88%	52.34%	96.84%	81.56%
Lok score	0.804	≤0.01	≥0.55	92.86%	58.98%	36.95%	95.24%	58.24%
Liaoning score	0.755	≤0.01	<0.55	77.38%	80.47%	56.52%	91.56%	79.71%
Splenic stiffness	0.858	≤0.01	≥31	89.29%	66.88%	41.67%	66.47%	76.47%

On multivariate logistic regression analysis, splenic stiffness (p < 0.001; odds ratio {OR}, 2.923; 95% CI, 1.62-5.3) and EVendo score (p = 0.026; OR, 1.084; 95% CI, 1.010-1.164) were independent predictors of HRV in patients with CLD (Table 5).

**Table 5:** Multivariate analysis of the non-invasive predictor of HRV HRV, high-risk varices; CI, confidence interval

Scores	P-value	Odds ratio	CI (95%)	
			Lower limit	Upper limit
Lok score	0.210	1.103	0.064	3.596
Liaoning score	0.669	1.187	0.542	2.599
Splenic stiffness	<0.001	2.923	1.62	5.3
EVendo score	0.026	1.084	1.010	1.164

## Discussion

This study evaluated and compared the efficacy of four non-invasive tools, Lok score, splenic stiffness, EVendo score, and Liaoning score, for predicting esophageal varices (EV) in patients with chronic liver disease (CLD) in a Pakistani cohort. Our findings revealed that all four scores showed statistically significant associations with the presence of EV. Among them, splenic stiffness (SS) emerged as the best-performing parameter in terms of AUROC, while EVendo and Liaoning scores demonstrated notable diagnostic accuracy and predictive strength.

In our study, SS exhibited the highest AUROC (0.858), aligning with previous literature that highlights its strong correlation with portal hypertension. In a meta-analysis by Ma et al. (2016), the diagnostic performance of SS was superior to liver stiffness in predicting esophageal varices (0.88 versus 0.81) with a higher sensitivity and specificity of 88% and 78%, respectively [14]. Our study showed a slightly higher sensitivity (89.29%) for SS at the cutoff of ≥31 kPa, which is comparable to the findings observed by Ma et al. [14]. However, the specificity of SS in our study was slightly lower (66.47%) and can be attributed to the difference in the demographics of the studied population with variable spleen sizes and fibrosis stages.

The Lok score, although originally designed for predicting cirrhosis, has been increasingly evaluated for its ability to predict EV [7]. Sungkar et al. demonstrated a good sensitivity of 74.7%, specificity of 72%, and diagnostic accuracy of 73.7% of Lok score in predicting EV in the Indonesian population [15]. In our study, we observed a high AUROC (0.804) of Lok score with an excellent sensitivity of 92.86%. However, it had a relatively lower specificity (58.98%) and diagnostic accuracy (58.24%) in the Pakistani population as compared to the Indonesian population. However, Afzal Tarar et al. demonstrated similar results of Lok score in predicting EV in the Pakistani population, with an excellent sensitivity and a limited specificity [7].

The EVendo score also performed robustly, with an AUROC of 0.76, high sensitivity (92.94%), and overall diagnostic accuracy of 81.56%. Jan et al. also showed an excellent sensitivity, specificity, and diagnostic accuracy of EVendo score in predicting EV in the Pakistani population [9]. Our findings affirm its reproducibility in South Asian populations, expanding its potential generalizability. Notably, EVendo was an independent predictor of high-risk varices (HRV) in our multivariate regression, further emphasizing its clinical relevance in high-stakes decision-making.

The Liaoning score, a relatively new model developed in a Chinese cohort, also showed satisfactory performance with an AUROC of 0.755 and diagnostic accuracy of 79.71% [11,13]. Despite originating in a genetically and etiologically distinct population, its effective performance in our study underscores its potential for broader application. However, the lack of independent predictive value for HRV in multivariate analysis suggests that it may function best in conjunction with other tools rather than as a sole predictor.

One important strength of our study is its comprehensive head-to-head comparison of multiple non-invasive tools in a single cohort of the Pakistani population, which is rare in existing literature. Moreover, the inclusion of a large and diverse sample of Pakistani patients with varied etiologies of CLD enhances the generalizability of our findings to similar resource-limited settings. However, certain limitations merit consideration. First, a single-centered study may limit the generalizability of the results to other populations. Second, splenic stiffness measurement requires specific equipment and technical expertise, which may not be readily available in all centers. Third, our cross-sectional design precludes the assessment of temporal changes in varices or progression. Lastly, endoscopic findings, though standardized, may still carry inter-observer variability.

## Conclusions

Our study showed that all these non-invasive indices are significant predictors of the presence of esophageal varices in patients with chronic liver disease in the Pakistani population. Of these, splenic stiffness had the highest diagnostic accuracy and was the most reliable predictor, whereas EVendo score had good sensitivity and ranked as an independent predictor of high-risk varices. These results imply that the use of non-invasive measures such as splenic stiffness and EVendo score in clinical practice can aid in the stratification of patients and decrease unnecessary endoscopy-based procedures, specifically in limited resource-based health facilities such as in Pakistan. However, further multicentered studies with large sample sizes are required to validate our results.
